# Patient and Parent Perspectives on Improving Pediatric Asthma Self-Management Through a Mobile Health Intervention: Pilot Study

**DOI:** 10.2196/15295

**Published:** 2020-07-03

**Authors:** Michelle Nichols, Sarah Miller, Frank Treiber, Kenneth Ruggiero, Erin Dawley, Ronald Teufel II

**Affiliations:** 1 College of Nursing Medical University of South Carolina Charleston, SC United States; 2 College of Medicine Medical University of South Carolina Charleston, SC United States

**Keywords:** asthma, mobile health, ecological momentary assessment, adolescents, medication adherence, self-management, mobile phone

## Abstract

**Background:**

Asthma is a common chronic pediatric disease that can negatively impact children and families. Self-management strategies are challenging to adopt but critical for achieving positive outcomes. Mobile health technology may facilitate self-management of pediatric asthma, especially as adolescents mature and assume responsibility for their disease.

**Objective:**

This study aimed to explore the perceptions of youths with high-risk asthma and their caregivers on the use of a smartphone app, Smartphone Asthma Management System, in the prevention and treatment of asthma symptoms, possible use of the app to improve self-management of asthma outside traditional clinical settings, and the impact of asthma on everyday life to identify potential needs for future intervention development.

**Methods:**

Key informant interviews were completed with parent-child dyads post participation in an asthma management feasibility intervention study to explore the perceptions of users on a smartphone app designed to monitor symptoms and medication use and offer synchronous and asynchronous provider encounters. A thematic qualitative analysis was conducted inductively through emergent findings and deductively based on the self-determination theory (SDT), identifying 4 major themes.

**Results:**

A total of 19 parent-child dyads completed the postintervention interviews. The major themes identified included autonomy, competence, relatedness, and the impact of asthma on life. The participants also shared their perceptions of the benefits and challenges associated with using the app and in the self-management of asthma. Both children and parents conveyed a preference for using technology to facilitate medication and disease management, and children demonstrated a strong willingness and ability to actively engage in their care.

**Conclusions:**

Our study included support for the app and demonstrated the feasibility of enhancing the self-management of asthma by youth in the community. Participant feedback led to intervention refinement and app improvements, and the use of the SDT allowed insight into motivational drivers of behavioral change. The use of mobile apps among high-risk children with asthma and their parents shows promise in improving self-management, medication adherence, and disease awareness and in reducing overall disease morbidity.

## Introduction

### Background

Asthma is one of the most common chronic pediatric conditions in the United States [1,2]. Asthma prevalence estimates highlight the racial disparities faced by minority youth, with higher prevalence among non-Hispanic (NH) black children (13.4%) compared with their NH white counterparts (7.8%) [1]. Higher percentages of minority children with asthma also have suboptimal asthma control [3]. Poorly controlled asthma results in substantial burden to youth and their caregivers as it negatively impacts the quality of life, school attendance, and parental missed workdays and increases mortality among youth [4-6]. Identifying mechanisms that can reduce the burden of asthma and improve symptom control through education, skill development, and accurate and consistent use of inhaler medications may reduce morbidity and mortality among children with high-risk asthma [7].

Asthma control among adolescents can be affected by numerous factors, including knowledge about self-management of asthma, self-efficacy, medication adherence, exposure to smoke and other triggers, and access to medical care. As youth transition to older adolescence, they typically become increasingly responsible for self-management of asthma, making this transitional period an important time to act as life-long health behaviors are established [7]. The increasing use of technology across all demographic groups has led to the proliferation of mobile health (mHealth) apps for the management of asthma. Despite expanded availability and use, gaps remain in the development and implementation of mobile technology to enhance the self-management of asthma [8,9]. Most apps are not rigorously developed with patient engagement or feedback [8,9], which is an important consideration when designing and evaluating digital interventions to meet the needs of patients with increased morbidity and mortality, such as high-risk asthma [10]. Recent studies on the use of mobile apps to promote self-management among youth with diabetes highlight the benefits and continued need for patient-centered design processes at each phase of the intervention design process [11,12]. A systematic review by Ramsey et al [13] exploring publicly available mobile apps for asthma management noted a continued need for efficacy testing of apps and for intervention design to be grounded in evidence-based strategies. In addition, few apps are developed or implemented using established theories for health behavior or behavioral change, which can decrease the likelihood of an app influencing the skill development and ultimately, the desired health behavior change [14,15]. To address this gap, self-determination theory (SDT) was used to inform the development and refinement of the app and served as the overarching framework for the postintervention qualitative data collection and analysis within this study. The members of this research team have successfully used SDT and other behavioral theories to guide app development and refinement in previous studies [16,17].

Within SDT, relatedness (specifically shared experience) has been associated with positive health behaviors in youth with asthma. A study by Geryk et al [18] explored the preferences of parents and clinicians for an mHealth app using an iPod Touch to promote self-management of asthma in adolescents. The results showed receptivity toward facilitating a 2-way asthma care communication approach; however, their study did not include the perspectives of youth with asthma. They highlighted the benefit of integrating a theoretical framework within the implementation research designs to increase adolescent self-management, medication adherence, and asthma control and suggested including the ability to tailor interventions according to participant feedback [18]. Recognizing the need to increase engagement with youth who have asthma, Sleath et al [19] studied the use of an asthma question prompt list for youth with asthma, which they found encouraged youth randomized to the interventional arm to get actively involved during clinic visits [19]. The objective of this study was to engage youth and adolescents with high-risk asthma and their caregivers via key informant interviews (KIIs) to explore their perceptions of the use of a smartphone app in the prevention and treatment of asthma symptoms and the potential increase of asthma self-management outside clinical settings. Our team worked with software developers to design and implement a Smartphone Asthma Management System (SAMS) app in 2 phases for youth with high-risk asthma. Phase 1 focused on assessing the feasibility and acceptability of collecting real-time asthma information, and phase 2 included the intervention of asynchronous video assessment of inhaler use techniques and synchronous direct-to-consumer telehealth encounters. Both phases included a qualitative assessment of the impact of asthma on daily life to better understand the unique experiences of participants and to further identify the potential needs of youth and parents of children with asthma. By identifying and understanding the larger scope of the impact of asthma on quality of life, motivational strategies to promote behavioral change and activation can be leveraged for future interventional expansion [20]. Soliciting input through initial feasibility testing of youth and their parents on the acceptability, feasibility, and adaptability of mHealth apps during all phases of development is critical to understand the potential long-term effectiveness and viability of using this technology in managing high-risk pediatric asthma. The results from our feasibility study will inform intervention modifications for a future full-scale randomized clinical trial.

### Theoretical Framework

Originating from the work of Deci and Ryan [21], the SDT explores factors that motivate individuals and allows insight into how to best tailor interventions that optimize action. Serving as a framework to understand and explore intrinsic and extrinsic motivation, the SDT can be used to explain factors that may drive health behaviors [22]. A central tenet of the SDT is the distinction of motivation, whether controlled or autonomous. Within a controlled motivational domain, individuals may feel a sense of obligation or duty to act in response to an employer or teacher’s directive, whereas within autonomous motivation, the individual acts fully on his or her own volition [21].

The SDT comprises 3 main constructs: autonomy, competence, and relatedness [21]. Autonomy allows for self-direction and personal choice on one’s actions and key decisions throughout life [21]. Competence includes self-efficacy and empowerment with skills (including health behaviors) as well as mastery over things important to the individual. Relatedness is the sense of belonging or connectedness one has to others, along with the feeling of being cared for [21]. Through recognition of each of these constructs, clinicians can develop interventions conducive to enhance self-motivation and self-management, in turn leading to improved health outcomes [21].

## Methods

### Study Sample

Children and adolescents, aged between 8 and 17 years, with high-risk asthma and their parents were invited to participate in our iterative design feasibility study to ensure that the final product is end user guided. The inclusion criteria were as follows: (1) patient with high-risk asthma, defined as a patient having an emergency department or hospital visit for asthma in the past 12 months or the child’s primary asthma provider in the outpatient setting having the opinion that the youth is at high risk for future asthma-related visits to the emergency department or hospital owing to the concern of noncompliance or poorly controlled asthma; (2) patient is prescribed a controller and rescue medication compatible with Bluetooth inhaler cap; (3) the caregiver or youth owns and is able to use a smartphone compatible with Bluetooth devices; (4) participant is English speaking; (5) patient with an identified primary care provider with at least one visit to the provider in the last year; and (6) patient with at least 1 identified caregiver present for enrollment, willingness, and availability to participate in study visits. This study was approved by the institutional review board of the Medical University of South Carolina. The participants were recruited from 2 children’s hospitals in South Carolina and through provider referrals to participate in our iterative design process. The qualitative data reported in this study represent all enrollees in both phases of the iterative design process.

### Mobile Health Platform

#### Iterative Design

The first phase of our iterative design included developing a process for real-time monitoring of the ecological momentary assessment (EMA) of asthma, asthma symptoms and other contextual factors such as emotional state, medication use via Bluetooth inhaler devices, and enrollee engagement via KII to inform the provider interactions in phase 2. The feasibility and acceptability data from phase 1 have been previously reported [23]. The second phase included the end user–guided provider interactions of asynchronous video capture of inhaler use techniques and synchronous direct-to-participant smartphone-based telehealth visits. Each study participant completed 1 video capture session and 1 telehealth visit during the intervention phase to assess feasibility, acceptability, and preferences toward these approaches, including their perceived benefits on the educational content from the personalized inhaler feedback received. Modifications to the data collection processes were made to include the collection of validated youth and parent surveys, including SDT metrics and removal of Bluetooth-enabled inhalers. The devices were removed for pragmatic reasons, including insufficient access to Bluetooth devices; initial participant and study team feedback regarding concerns over the accuracy of Bluetooth data; and their potential to add to the technical complexity for phase 2 enrollees, whom we wanted to focus on the newly developed provider interactions. The participants received SMS text message reminders to encourage completion of the EMA questions, video capture, and participation in the telehealth visit. Throughout the iterative design, our team considered the importance of developing competency (eg, the ability of youth to recognize symptoms and develop inhaler use techniques), autonomy (eg, telehealth visits performed by an educator trained in motivational interviewing), and relatedness (eg, encouraging but not requiring parental involvement in asthma management and developing remote provider encounters).

#### Real-Time Asthma Assessment

All enrollees had the SAMS app downloaded to their personal mobile phones and were requested to report EMAs daily. The EMA included 8 questions on their asthma symptoms, mood, or affect. Participants were asked to track asthma medication use, including controller and rescue inhalers, for a 2-month feasibility intervention period. EMAs reduce recall bias through real-time daily response reporting [24]; however, the frequency of response entries has the potential to increase participant burden [25]. All enrollees could view data on recorded medication use over their smartphones. Enrollees 1 to 14 recorded medication use with Bluetooth inhaler caps, and enrollees 15 to 22 recorded medications manually. Reminders were sent if real-time assessment data were missing for 3 days. Further details on the development, feasibility, and acceptability of the real-time assessments on SAMS are reported elsewhere [23].

#### End User–Guided Provider Interactions

Provider interactions, including asynchronous video capture of inhaler use technique and synchronous direct-to-participant smartphone-based telehealth visits, were discussed with all enrollees during their interviews. As the iterative design process progressed incorporating participant feedback, the protocols and programming were developed and refined for enrollees 14 to 22 to participate in these provider interactions during their 2-month feasibility trial. The enrollees were requested to participate at least once in both provider interactions over the 2-month study period. The asynchronous inhaler use technique was performed via the enrollee’s smartphone by a tab within the SAMS app. Once activated, the enrollee would receive on-screen prompts regarding inhaler use technique, with a counter designed to be consistent with national guidelines. The video was encrypted and transferred to a secure server for review by an asthma educator who was a respiratory therapist, with feedback provided via SMS text, during the telehealth visit, or both. Synchronous telehealth visits were performed by enrollees using an embedded link to Vidyo, a telehealth video conferencing system, present in the SAMS app. An asthma educator trained in motivational interviewing performed these visits. Telehealth visits were requested at week 4 of the study and could be performed at a convenient time for enrollees.

### Data Collection and Analysis

Following the completion of the 2-month interventional phase of the study, dyadic KIIs were conducted with the enrolled youth and caregivers using a semistructured interview guide (Table 1). The interview guide was structured to explore the constructs of the SDT and to identify the perceptions of participant’ on the challenges and benefits associated with SAMS, including the potential burden associated with the use of EMA at home and in the community. Participants were also asked about the impact of asthma on their daily lives. Using an iterative interventional design approach, 2 waves of participants were enrolled. Data from phase 1 were collected, analyzed, and used to refine the intervention content and delivery for phase 2. Participants from both phases were invited to complete postintervention KIIs. The validated survey tools of youth and caregivers were collected in phase 2 and focused on asthma control, medication adherence, and SDT constructs (eg, self-efficacy, which is akin to competence) to better understand the study population and change of the measure from enrollment to the final visit.

KIIs were audio recorded, professionally transcribed, and reviewed for accuracy. The transcripts were then uploaded to NVivo 11.4.1 for data analysis. An experienced qualitative research methodologist analyzed the transcripts using a thematic content analysis approach, allowing identification of implicit and explicit themes found in the data [26-28]. A theoretical framework was used to situate findings, and researchers created a codebook containing operational definitions and examples, which enabled a systematic approach to data analysis and interpretation [26,29]. Data were deductively coded according to the SDT and the challenges and benefits of SAMS and then inductively through emergent codes [30]. Following line-by-line coding of raw text, the results were confirmed by members of the research team for confirmability of interpretation [31]. An audit trail of data coding and interpretation was maintained to increase the trustworthiness of the findings [32].

**Table 1:** Key informant interview guide.

Objective	Parent-directed interview prompts	Child-directed interview prompts	Dyad-directed interview prompts (parent and child)
Background information	Tell me about his/her asthma medications before joining this study. What was working and what wasn’t? How about now that he/she has been in the research study? What has changed?	Can you tell me how old you are, what grade you are in, and whether you have your own cell phone or not? If not, whose cell phone did you use for this study? About how many kids in your class at school would you say have their own cell phones? Do most kids your age have them, some, or only a few?	N/A^a^
Impact of asthma on life	What is it like for you having a child with asthma? How does your child having asthma affect your work? What about you, (parent/caregiver), how has his/her asthma affected your schedule?	What is it like to live with asthma? How does your asthma affect your ability to join in on activities with your friends and family?	How often would you say you have missed school or work because of your child’s asthma in the past? What about now?
Perceptions of SAMS^b^	What about the system helped you manage your child’s asthma? What about the system made it difficult?	N/A	Tell me about how comfortable you were using the SAMS system. If you could describe the SAMS system to someone, what would you say about it? Why? Now with this system, there is very little human interaction because you receive text messages or alerts versus actually speaking to someone. What are your thoughts on this? Which questions that were sent through the messages were helpful? Which ones were annoying? How could we make the messages better? What was the best thing about the SAMS system? What about the worst thing?
Recommendations	Some parents have suggested receiving feedback on how well their children are doing with their inhalers. What are your thoughts on this? What types of things do you think would be helpful to know? If you were to pick an ideal scenario for support on managing your child’s asthma, what would you look for? Would you want mostly to use technology or combine it with telephone calls? If combined, how frequently would be the right amount of time for telephone calls?	Some people have suggested we add a type of game in the system to make it more fun. What do you think about that? When you play electronic games, what makes them the most fun? What types of rewards do you like to receive on the games when you reach certain levels?	Tell me what would have made this easier to use or what you would change about it if you could.

^a^N/A: not applicable.

^b^SAMS: Smartphone Asthma Management System.

## Results

### Sample Characteristics

A total of 19 parent-child dyads (overall mean age of the children was 10.89 years, SD 2.73) participated in the postintervention interviews from the original 22 dyads who completed the study intervention. Furthermore, 3 additional children completed the study procedures (phase 1), which included a 60-day data capture, but were lost to follow-up and unable to be reached for inclusion in the postintervention interview; thus, they were not included in the qualitative analysis. Overall, 7 of the 19 who completed the study intervention were female. Participants (N=22) reported insurance coverage as Medicaid (18/22, 82%), self-paid (3/22, 14%), and private insurance (1/22, 5%). Most (n=16) of the participants were African American (84%), with 2 children identifying as mixed race and 1 child as white. Phase 1 included 11 participants, mean age of children was 10.9 years (SD 2.73), followed by an additional 8 participants in phase 2, mean age of children was 12 years (SD 3.02). Phase-specific demographic data are presented in Table 2, and the child and parent survey tools used in phase 2 are highlighted in Table 3.

**Table 2:** Study population demographics by design phase.

Demographics			Phase 1 (n=14), n (%)	Phase 2 (n=8), n (%)
**Age (years)**				
	8-10		9 (64)	4 (50)
	11-13		3 (21)	2 (25)
	14-16		2 (14)	2 (25)
**Gender**				
	Male		5 (36)	6 (75)
	Female		9 (64)	2 (25)
**Race**				
	White		0 (0)	6 (75)
	Black		13 (93)	1 (13)
	Other		1 (7)	1 (13)
**Ethnicity**				
	Hispanic		0 (0)	0 (0)
	Non-Hispanic		14 (100)	8 (100)
**Insurance**				
	Medicaid		11 (79)	7 (88)
	Private		1 (7)	0 (0)
	Self-paid		2 (14)	1 (13)
**Clinical characteristics**				
	**Asthma severity**			
		Intermittent	0 (0)	0 (0)
		Mild persistent	1 (7)	0 (0)
		Moderate persistent	8 (57)	7 (88)
		Severe persistent	3 (21)	0 (0)
	**Asthma control**			
		Well controlled	0 (0)	0 (0)
		Not well controlled	12 (86)	7 (88)
		Very poorly controlled	1 (7)	1 (13)

**Table 3:** Child and parent survey tools used in development phase 2 (n=8).

Survey tool	Enrollment, mean (SD)	Final 2-month visit, mean (SD)	*P* value
Asthma control test^a^	17.4 (3.4)	21.6 (2.3)	.01
Child asthma belief scale	3.6 (0.8)	4.3 (0.6)	.06
Reported medication adherence survey	3.8 (0.7)	4.2 (0.7)	.30
Parental asthma self-efficacy scale	4.2 (0.5)	4.4 (0.3)	.19
Parental belief in treatment efficacy	4.7 (0.2)	4.5 (0.6)	.39

^a^*P* value less than .05 enrollment compared with final visit controlling for patient as a random effect.

### Impact of Asthma on Life

Parents and children alike voiced concerns over the impact of asthma on their daily lives. Parental concerns ranged from financial burdens, loss of time from work, challenges in managing busy schedules, and an overwhelming sense of fear and uncertainty related to asthma exacerbations and what that means for their children’s well-being. Children explained how asthma affects their ability to engage in activities, causes them to miss school days, and can even lead to embarrassment and potential stigma from being considered *different* from other children. Exemplar quotes are presented in Textbox 1.

**Exemplar quotes on the impact of asthma on daily life.**

**Activities**

- “I can’t always run as long as I want.” [Child no. 6, phase 2]
- “There are certain things I can’t do like strenuous exercises or certain sports.” [Child no. 8, phase 1]
- “If it’s something really active, I can’t do it as much as everybody else does...I tried to join the dance team, but some of the stuff they do—they do a lot of stuff—so I’ll get tired faster than everybody else.” [Child no. 12, phase 1]
- “Like when you play basketball, you can only play for a certain amount of time.” [Child no. 4, phase 2]
- “Actually, when I am running the bases my chest feels tight.” [Child no. 9, phase 1]
- “It’s kind of hard because I can’t do certain stuff anymore that I used to do.” [Child no. 7, phase 2]

**School**

- “Missed maybe four days within the last year or so.” [Parent no. 12, phase 1]
- “Sometimes at PE [physical education], when we’re like running around and I was in the corridor, my lungs started to hurt.” [Child no. 2, phase 1]
- “Missed only one week.” [Child no. 9, phase 1]
- “I think it was two or three days he was out.” [Parent no. 11, phase 1]
- “I missed a little bit of school a couple of weeks ago because I had a flare-up.” [Child no. 8, phase 2]
- “A month...maybe five days.” [Parent no. 6, phase 2]

**Family life**

- “Asthma affects our life daily because it just impacts everything. When he has an asthma attack, I miss work, he misses school, my mom is responsible for getting my daughters to school because he is in the hospital. That is when it is not controlled.” [Parent no. 1, phase 1]
- “I have two smaller children and that [hospitalizations] kind of pulls you away from the two that you have at home or stuff you have to do for the younger kids or other kids.” [Parent no. 12, phase 1]
- “We don’t turn the heat on overnight because it makes him cough. I was like ‘girls, bundle up because either he can breathe and you’re cold or he can’t breathe’.” [Parent no. 2, phase 2]

**Work**

- “If the school calls, I got to leave work and go pick her up.” [Parent no. 4, phase 2]
- “While he was in the hospital [Dad] missed the first day and a half [of work] wanting to make sure everything was okay with him.” [Parent no. 9, phase 1]
- “Sometimes when he is in distress and all and has to go to the hospital because of his asthma, we both [parents] sometimes miss work. I have to do what I have to do for my child.” [Parent no. 8, phase 2]
- “At work, I’m a supervisor, I’m with patients ‘cause I’m in the health care field so I can’t just up and leave the patients, but I also have a child that I have to worry about. That comes first. I have to take a lot of days off.” [Parent no. 6, phase 2]

### Autonomy

As children age and mature, there is a natural tendency for them to want to self-manage their health and health care needs, which supports their intrinsic motivation to be more independent [33]. This desire is often juxtaposed with parental needs to ensure optimal health outcomes for their children and the difficulties they face in relinquishing control of day-to-day disease management. Parental concerns can stem from fear associated with asthma exacerbations and the safety and well-being of their children. One parent (phase 2) shared that she just recently allowed her 8-year-old son to start sleeping in his own room because she was afraid that he may struggle with breathing during the night and she might not be able to respond in a timely manner. The experience of watching their child go through an asthma crisis brought about significant fear for many parents, reducing their comfort level in allowing their child to self-manage their disease:

Just the thought of not being able to breathe—it’s like drowning and you are not in water or something. It’s just terrifying sound to me.

Parent no. 2, phase 2

Sometimes it can be a little scary, especially if she is having difficulty breathing and not verbalizing it...you have to look out for certain signs like if they are gasping for air or like if their breathing pattern has changed or increased or decreased.

Parent no. 1, phase 2

It’s very scary...when [he] first came home from the hospital, I was nervous. I watched him all the time sleeping and if I hear a cough, I get so nervous...I have to monitor and make sure that he’s taking his medicine and getting this right.

Parent no. 11, phase 1

When he had to be flown to the hospital for his asthma flare-up, that was pretty scary.

Parent no. 8, phase 2

As part of the study intervention, participants received reminder notifications on medication administration and survey completion. Participants conveyed strong support for the reminder alerts in facilitating autonomy among children and in transitioning from parent-managed to shared responsibility. Older adolescents with access to their own mobile devices expressed that they were able to take on a more active role and expanded autonomy through self-management of medication administration and symptom management. One parent shared how her 15-year-old daughter “knows more about what she is dealing with” now that she is older and that “she is self-managing” (Parent no. 12, phase 1). Another parent indicated that her 10-year-old daughter (phase 2), who was using the parent’s phone, would take the initiative, ask for the phone, and would remind the mother that she had to record her medications and answer the questions. Even children as young as 8 years old demonstrated the ability and desire to self-manage their disease. One mother conveyed how her son would state “this is private” when related to the responses he was entering for the questions, as they pertained to his personal health (Parent no. 2, phase 2) and he wanted to take responsibility for the entries:

It helps me. Like it reminds me that I need, in case I forget to take my medicine or anything, it reminds me to stop whatever I’m doing and go take my medicine.

Child no. 1, phase 2

Now that she is older, I let her do it...it teaches them responsibility...That gives them a little feel good. I’m doing something. They’re more in control like that...it makes them feel good about themselves, too.

Parent no. 8, phase 1

I try to allow him to do it more than myself, so when he wasn’t around, then I didn’t do it ‘cause I wanted him to be responsible to do it.

Parent no. 6, phase 2

I prefer the app because you could actually do it by yourself.

Child no. 8, phase 1

### Competence

Knowledge, understanding, and prior experience with asthma and asthma care management factored into participant responses:

Well, it’s been a little bit of a learning curve for me just because I’ve never had to deal with anyone with asthma before.

Parent no. 3, phase 2

We may think that they’re actually doing it correct, but if we’re not taught, then we don’t know how to tell them to correct stuff that they’re doing.

Parent no. 1, phase 2

Participants expressed a strong comfort level with the SAMS system following the initial demonstration, with several sharing similar feedback that it was quite easy to use. All participating children shared the sentiment that it was easy to use and highlighted the use of technology as a normative component of their daily lives:

I’m a kid, so the phone is my life.

Child no. 12, phase 1

I think that it’s good because like I like computers and stuff and I like to be on the phone a lot.

Child no. 8, phase 1

The features that contributed to the increased competence in managing asthmatic symptoms included educational content and the ability to tailor feedback following the video capture of the use of inhaler. For the video capture of the inhaler use technique, participants were prompted with a series of on-screen prescripted prompts to promote proper inhaler use techniques. Tailored feedback was provided by the respiratory therapist following a review of the video capture feature within the app. The tailored feedback included tips on proper inhalation techniques such as mouth placement on the inhaler, holding one’s breath after inhalation, and shaking the inhaler before use. This was particularly true for 1 newly diagnosed child, as he initially ran out of medication early because of improper technique. Others describe the benefit of capturing inhaler use for technique refinement and parental ability to ensure proper medication administration adherence:

He didn’t know how to really use it. He’s new to this, so he just pumping away and thinking he-he pumped the first time, it’s not going in, so we do it again and keep on trying till get it. I think that’s why he ran out so fast.

Parent no. 11, phase 1

I was able to use the help option there within the app, and it gave me some directives. I was able to figure it out pretty quickly.

Parent no. 9, phase 1

I think that’s good because you can learn how to take your medicine better so that it’ll work better.

Child no. 8, phase 1

The tool to me is super easy for somebody that’s new to having a child with asthma. It really did help me...it’s walking you through—this is exactly how you are to do it.

Parent no. 3, phase 2

The participating children shared a sense of pride and accomplishment in self-managing their care, an essential factor in enhanced competence, which can facilitate increased engagement:

I felt really comfortable. It was an easy app to learn and I’ve been using it.

Child no. 3, phase 2

[I was] pretty comfortable ‘cause it’s like I knew I took my medicine and...it helps you remember to take your meds...I didn’t wanna get sick.

Child no. 6, phase 2

### Relatedness

Children reported the importance of relatedness, underscoring the importance of peer relationships and social acceptance during childhood and adolescent development. They further expressed how asthma has the potential to increase social isolation and limit the ability to engage with peers at the same level or for the same duration (Textbox 1). Several articulated concerns over being perceived as *different*, a strong desire to be considered *normal*, and as preferring to maintain privacy regarding the awareness of their disease with others. An example of this was highlighted by an 8-year-old boy who became very upset when using the inhaler and answering questions at school as his classmates “didn’t even know I have asthma” and once realized, children noticed and began talking:

But once I did it at school because she came and had lunch with me and then people were looking at it and I was mad.

Child no. 2, phase 2

Feeling supported and cared for by others were strong attributes of relatedness throughout the intervention and, in general, participants appreciated concern over health and well-being conveyed by others while participating in the study. One mother commented that it was good “to get to interact with the person on the other side” when referencing the video interaction and personal connectedness using mobile technology (Parent no. 4, phase 2). Others expressed appreciation in knowing that providers were actively involved and concerned with their child’s well-being and care management:

With the continual feedback or reminders, it actually made me aware that somebody was, in fact, watching.

Parent no. 1, phase 2

[The intervention] reassured me that there is genuine concern about how my son is doing and how he is recovering and whatever other treatments he may need.

Parent no. 9, phase 1

A lot of people in my class, when they heard that I was in the hospital because of my asthma, they said that they were, like, worried about me...it made me feel good.

Child no. 3, phase 2

### Challenges of Smartphone Asthma Management System

Although there was unanimous support for SAMS among all interviewees, some expressed challenges. The time required for video uploads was problematic for some, especially when waiting to upload one video before administering a second dose. One mother described how it was somewhat awkward for her son to hold the phone while capturing the video and simultaneously self-administering medication. She also shared that her smartphone would “time-out” and necessitate touching the screen, which would interrupt the video.

Bluetooth connection and proximity to the smartphone yielded challenges for families when the child did not have his or her own phone. This was exemplified further when children would be away from home for a period and data capture would be delayed. The exemplar quotes are presented in Table 4.

**Table 4:** Exemplar quotes on the benefits and challenges of Smartphone Asthma Management System.

Role	Benefits	Challenges
Parent	“Half the times I have so much going on, I forget. Whereas with the app, it it’s not taken, it pops up on the phone...and this way, it makes it much easier as far as controlling how she takes her medicine.” [Parent no. 12, phase 1] “It was helping me to remind her to take her medication...’cause the alarm goes off so for her to take her medicine and for her to answer the questions, so it’s like a double reminder in the evening when we are so busy.” [Parent no. 7, phase 1] “I think the text messages and alerts, not actually having to sit on the phone with a human and the hold times, the wait times—technology has taken over. I think it’s just so much more simpler.” [Parent no. 12, phase 1] “I don’t mind talking to people, but I don’t want you to call me every day. You know, text me every day is fine. You send me a message every day it’s fine, but I’m more of a texter in general than I am a talker.” [Parent no. 2, phase 2] “That was helpful because...she was kind of doing it wrong. So, when we talked to her, she kind of told us, you know, certain ways you’re supposed to do it and count. That was real helpful.” [Parent no. 4, phase 2] “I would ask her...‘oh, what type of feedback did you get in the video?’ and she expressed that she was told to sit up straight and I think something to do with the spacer.” [Parent no. 1, phase 2]	“So, you are sitting there for like four or five minutes because you take your second puff to get, you know, to film that...it was just a little difficult.” [Parent no. 3, phase 2] “I would have to site my phone in a neutral location or she would have to get closer to the room in order for it to pick up. As far as I was concerned, that’s the only flaw. Just like I said, she had went out for the weekend and wasn’t home. So, the phone doesn’t pick up if it’s not her phone and it’s not where she is. That for me is the only issue I have. Other than that, it was good.” [Parent no. 12, phase 1]
Child	“It helps you remember to take your meds.” [Child no. 6, phase 2]	N/A^a^

^a^N/A: not applicable.

### Benefits of Smartphone Asthma Management System

A recurring theme throughout the interviews was the benefit of reminders on medication adherence. Although intentions were strong to follow medical advice, the realities of balancing demanding schedules and daily responsibilities pose challenges with actual adherence. Numerous quotes supported the advantage of SAMS in facilitating improved medication adherence (Table 4).

Convenience was another benefit shared by the participants. Parents articulated how they often struggle to manage work and family responsibilities and how finding time to have a conversation during the work day with a provider can be challenging; however, they felt that SAMS facilitated communication in a convenient manner for all parties. Moreover, parents felt that SAMS improved day-to-day care management and reduced unplanned provider visits, indicative of fewer asthmatic exacerbations for the children and the ability for parents to reduce time lost from work (Table 4).

The use of technology to increase medication adherence and accuracy of medication administration was supported by children and parents. Technology was also embraced as a means to facilitate more immediate communication and assessment and as a mechanism for timely and targeted intervention. In addition, the use of technology, whether via messaging or through video interaction, was strongly preferred by most participants as the primary means of routine communication and interaction. None of the participants had concerns over any potential burden associated with the frequency of EMA. Although text messaging and video conferencing were valued means of interaction, none of the participants wanted to completely replace traditional face-to-face encounters.

## Discussion

### Principal Findings

The results of this study indicate that children with high-risk asthma and their parents prefer to use technology to facilitate medication administration and disease management. Although parents still want the ability to personally interact with clinicians on an as-needed basis, children and parents articulated an inclination toward the use of technology to manage routine maintenance and monitoring. Interestingly, parents did not have safety or privacy concerns with the use of video recordings of their children using inhalers. This may be related to the ready availability of access to clinicians and support provided with minimal impact on work and school schedules, which likely indicates that any potential privacy concerns were outweighed by the benefits of the intervention. Further exploration of this is warranted among more socioeconomically diverse populations.

In addition, the use of Bluetooth-enhanced mHealth apps may be a viable mechanism for optimizing youth-parent-clinician engagement in the management of children with high-risk asthma. As technology has become a normative part of life for children and parents in the United States, capitalizing on innovative strategies to improve self-management of chronic disease among high-risk populations and reduce disease burden is a natural step forward. Although our study focused specifically on SAMS, the knowledge gained from this study may be transferable to other apps and mobile platforms.

Our research was strengthened through the engagement of children and adolescents with asthma and their parents. We demonstrated that children, as the end users of mobile apps, are willing and able to participate in the self-management of their asthma and welcomed the use of technology to facilitate disease and symptom management, allowing for increased autonomy and responsibility of care. There were no concerns over the frequency of responding with EMA, although further study is needed to identify if this would remain consistent with longer intervention periods. Understanding the preferences of children and parents is crucial in translational research efforts, allowing providers to best understand what works and may not work for patients outside clinical settings and living in the community, and it also has relevance for interventional research design and clinical practice.

Although conducting dyadic interviews with parents and children may have influenced the children’s responses, it was important to understand the preferences and experiences of both parents and children following the use of the SAMS app. All children actively contributed during the KIIs, and interview questions were not sensitive in nature, thus minimizing the likelihood of child participants withholding thoughts because of parental presence. Including children and parental input in an iterative mobile app design process can enhance the likelihood of adaptation and adherence in clinical trials and allows clinicians to more broadly understand the preferences and practices of managing chronic pediatric illnesses, which can be translated into practice recommendations to improve clinical outcomes.

Our study, through the assessment of the impact of asthma on life, builds upon recommendations to include quality of life assessments as an integral component of asthma interventions designed to enhance adherence [34]. Understanding how asthma affects day-to-day life for children and families provides insight into factors that may serve as motivational drivers for this population by tailoring mHealth interventions by using personalized priorities to promote behavior change. Incorporating input from end users enables a process of co-designing the app and intervention between clinical and academic researchers and children with asthma and their parents. In addition, the use of the SDT as a theoretical framework proved effective, allowing insight into motivational drivers of behavioral change, knowledge levels and skill acquisition, and factors related to one’s need for interconnectedness in personal relationships. Both mobile apps and the use of SDT can facilitate the understanding and management of children with high-risk asthma residing in community settings. Furthermore, this study helps fill an identified gap in mobile app development as many have not used models of health behavior change in app design and content development.

### Strengths and Limitations

This study has several limitations. Our study design was informed by a wide range of causal models; however, a more structured approach to causal factors and exploration of mechanisms to support self-management through the app is recommended for future studies. In addition, in this iterative feasibility pilot study, we chose not to script out the roles within the dyads and focused on the natural process of the dialog between the parent and child. It may be beneficial to explore relational interactions when considering the interplay of interpersonal relationships and self-management. In future studies, we intend to include measures of motivation and other SDT constructs. Although our study included mechanisms to interact with providers and captured EMA data, the app currently lacks an automated interface with electronic medical records, a noted gap among apps designed to manage asthma [13]. Study recruitment via provider referrals may have enabled providers to refer patients with a higher likelihood of interest in mHealth or those who providers felt needed better asthma management. Furthermore, another limitation was the discontinuation of objective adherence monitoring via Bluetooth. Although this report focused on the postintervention qualitative perspectives of participating children and parents, it is worthwhile to note the limitation of not including passive adherence monitoring in both phases of the actual pilot intervention.

## Abbreviations

EMA: ecological momentary assessment
KII: key informant interview
NH: non-Hispanic
SAMS: Smartphone Asthma Management System
SDT: self-determination theory
